# Training nurses in an international emergency medical team using a serious role-playing game: a retrospective comparative analysis

**DOI:** 10.1186/s12909-024-05442-x

**Published:** 2024-04-22

**Authors:** Hai Hu, Xiaoqin Lai, Longping Yan

**Affiliations:** 1 Emergency Management Office of West China Hospital, Sichuan University, The street address: No. 37. Guoxue Road, Chengdu City, Sichuan Province China; 2 China International Emergency Medical Team (Sichuan), Chengdu City, Sichuan Province China; 3 Emergency Medical Rescue Base, Sichuan University, Chengdu City, Sichuan Province China; 4 Day Surgery Center, West China Hospital, Sichuan University, Chengdu City, Sichuan Province China; 5 Department of Thoracic Surgery, West China Tianfu Hospital, Sichuan University, Chengdu City, Sichuan Province China; 6 West China School of Nursing, Sichuan University, Chengdu City, Sichuan Province China; 7 West China School of Public Health, Sichuan University, Chengdu, Sichuan China; 8 West China Fourth Hospital, Sichuan University, Chengdu, Sichuan China

**Keywords:** Rescue work, Gamification, Simulation training

## Abstract

**Background:**

Although game-based applications have been used in disaster medicine education, no serious computer games have been designed specifically for training these nurses in an IEMT setting. To address this need, we developed a serious computer game called the IEMTtraining game. In this game, players assume the roles of IEMT nurses, assess patient injuries in a virtual environment, and provide suitable treatment options.

**Methods:**

The design of this study is a retrospective comparative analysis. The research was conducted with 209 nurses in a hospital. The data collection process of this study was conducted at the 2019-2020 academic year. A retrospective comparative analysis was conducted on the pre-, post-, and final test scores of nurses in the IEMT. Additionally, a survey questionnaire was distributed to trainees to gather insights into teaching methods that were subsequently analyzed.

**Results:**

There was a significant difference in the overall test scores between the two groups, with the game group demonstrating superior performance compared to the control group (odds ratio = 1.363, *p* value = 0.010). The survey results indicated that the game group exhibited higher learning motivation scores and lower cognitive load compared with the lecture group.

**Conclusions:**

The IEMT training game developed by the instructor team is a promising and effective method for training nurses in disaster rescue within IEMTs. The game equips the trainees with the necessary skills and knowledge to respond effectively to emergencies. It is easily comprehended, enhances knowledge retention and motivation to learn, and reduces cognitive load.

**Supplementary Information:**

The online version contains supplementary material available at 10.1186/s12909-024-05442-x.

## Background

Since the beginning of the twenty-first century, the deployment of international emergency medical teams in disaster-stricken regions has increased world wide [1]. To enhance the efficiency of these teams, the World Health Organization (WHO) has introduced the International Emergency Medical Team (IEMT) initiative to guarantee their competence. Adequate education and training play a vital role in achieving this objective [2].

Nurses play a vital role as IEMTs by providing essential medical care and support to populations affected by disasters and emergencies. Training newly joined nurses is an integral part of IEMT training.

Typical training methods include lectures, field-simulation exercises, and tabletop exercises [3–5]. However, lectures, despite requiring fewer teaching resources, are often perceived as boring and abstract. This may not be the most ideal method for training newly joined nurses in the complexities of international medical responses. However, simulation field exercises can be effective in mastering the knowledge and skills of disaster medicine responsiveness. However, they come with significant costs and requirements, such as extended instructional periods, additional teachers or instructors, and thorough preparation. These high costs make it challenging to organize simulation exercises repeatedly, making them less ideal for training newly joined nurses [6].

Moreover, classic tabletop exercises that use simple props, such as cards in a classroom setting, have limitations. The rules of these exercises are typically simple, which makes it challenging to simulate complex disaster scenarios. In addition, these exercises cannot replicate real-life situations, making them too abstract for newly joined nurses to fully grasp [7, 8].

Recently, game-based learning has gained increasing attention as an interactive teaching method [9, 10]. Previous studies have validated the efficacy of game-based mobile applications [11, 12]. Serious games that align with curricular objectives have shown potential to facilitate more effective learner-centered educational experiences for trainees [13, 14]. Although game-based applications have been used in disaster medicine education, no serious computer games have been designed specifically for training newly joined nurses in an international IEMT setting.

Our team is an internationally certified IEMT organization verified by the WHO, underscoring the importance of providing training for newly joined nurses in international medical responses. To address this need, we organized training courses for them. As part of the training, we incorporated a serious computer game called the IEMTtraining game. In this game, players assume the roles of IEMT nurses, assess patient injuries in a virtual environment, and provide suitable treatment options. This study aims to investigate the effectiveness of the IEMTtraining game. To the best of our knowledge, this is the first serious game specifically designed to train newly joined nurses in an IEMT setting.

The IEMTtraining game was subsequently applied to the training course for newly joined nurses, and this study aimed to investigate its effectiveness. To the best of our knowledge, this is the first serious game specifically designedto train newly joined nurses in an IEMT setting.

## Method

### Study design

This study was conducted using data from the training records database of participants who had completed the training. The database includes comprehensive demographic information, exam scores, and detailed information from post-training questionnaires for all trainees. We reviewed the training scores and questionnaires of participants who took part in the training from Autumn 2019 to Spring 2020.

The local Institutional Review Committee approved the study and waived the requirement for informed consent due to the study design. The study complied with the international ethical guidelines for human research, such as the Declaration of Helsinki. The accessed data were anonymized.

### Participants

A total of 209 newly joined nurses needed to participate in the training. Due to limitations in the size of the training venue, the trainees had to be divided into two groups for the training. All trainees were required to choose a group and register online. The training team provided the schedule and training topic for the two training sessions to all trainees before the training commenced. Each trainee had the opportunity to sign up based on their individual circumstances. Furthermore, the training team set a maximum limit of 110 trainees for each group, considering the dimensions of the training venue. Trainees were assigned on a first-come-first-served basis. In the event that a group reached its capacity, any unregistered trainees would be automatically assigned to another group.

In the fall of 2019, 103 newly joined nurses opted for the lecture training course (lecture group). In this group, instructors solely used the traditional teaching methods of lectures and demonstrations. The remaining 106 newly joined nurses underwent game-based training (game group). In addition to the traditional lectures and demonstrations, the instructor incorporated an IEMTtraining game to enhance the training experience in the game group.

### The IEMTTraining game

The IEMTtraining game, a role-playing game, was implemented using the RPG Maker MV Version1.6.1 (Kadokawa Corporation, Tokyo, Tokyo Metropolis, Japan). Players assumed the roles of rescuers in a fictional setting of an earthquake (Part1 of Supplemental Digital Content).

The storyline revolves around an earthquake scenario, with the main character being an IEMT nurse. Within the game simulation, there were 1000 patients in the scenario. The objective for each player was to treat as many patients as possible to earn higher experience points compared to other players. In addition, within the game scene, multiple nonplayer characters played the role of injured patients. The players navigate the movements of the main character using a computer mouse. Upon encountering injured persons, the player can view their injury information by clicking on them and selecting the triage tags. The player can then select the necessary medical supplies from the kit to provide treatment. Additionally, the player is required to act according to the minimum standards for IEMTs, such as registration in the IEMT coordination cell and reporting of injury information following the minimum data set (MDS) designed by the WHO [15, 16]. This portion of the training content imposes uniform requirements for all IEMT members, hence it is necessary for IEMT nurses to learn it. All correct choices result in the accumulation of experience points. Game duration can be set by the instructor and the player with the highest experience points at the end of the game.

### Measurement

We have collected the test scores of the trainees in our training database to explore their knowledge mastery. Additionally, we have collected post-training questionnaire data from the trainees to investigate their learning motivation, cognitive load, and technology acceptance.

### Pre-test, post-test, and final test

All trainees were tested on three separate occasions: (1) a “pre-test”before the educational intervention, (2) a “post-test”following the intervention, and (3) a “final test”at the end of the term (sixweeks after the intervention). Each test comprised 20 multiple-choice questions (0.5 points per item) assessing the trainees’ mastery of crucial points in their knowledge and decision-making. The higher the score, the better the grade will be.

### Questionnaires

The questionnaires used in this study can be found in Part 2 of the Supplemental Digital Content.

The learning motivation questionnaire used in this study was based on the measure developed by Hwang and Chang [17]. It comprises seven items rated on a six-point scale. The reliability of the questionnaire, as indicated by Cronbach’s alpha, was 0.79.

The cognitive load questionnaire was adapted from the questionnaire developed by Hwang et al [18]. It consisted of five items for assessing “mental load” and three items for evaluating “mental effort.” The items were rated using a six-point Likert scale. The Cronbach’s alpha values for the two parts of the questionnaire were 0.86 and 0.85, respectively.

The technology acceptance questionnaire, which was only administered to the game group, as it specifically focused on novel teaching techniques and lacked relevance tothe lecture group, was derived from the measurement instrument developed by Chu et al [19]. It comprised seven items for measuring “perceived ease of use” and six items for assessing “perceived usefulness.” The items were rated on a six-point Likert scale. The Cronbach’s alpha values for the two parts of the questionnaire were 0.94 and 0.95, respectively.

### Procedure

The lecture group received 4 hours of traditional lectures. Additionally, 1 week before the lecture, the trainees were provided with a series of references related to the topic and were required to preview the content before the class. A pre-test was conducted before the lecture to assess the trainees’ prior knowledge, followed by a post-test immediately after the lecture, and a final test 6 weeks after training.

**Fig. 1:**
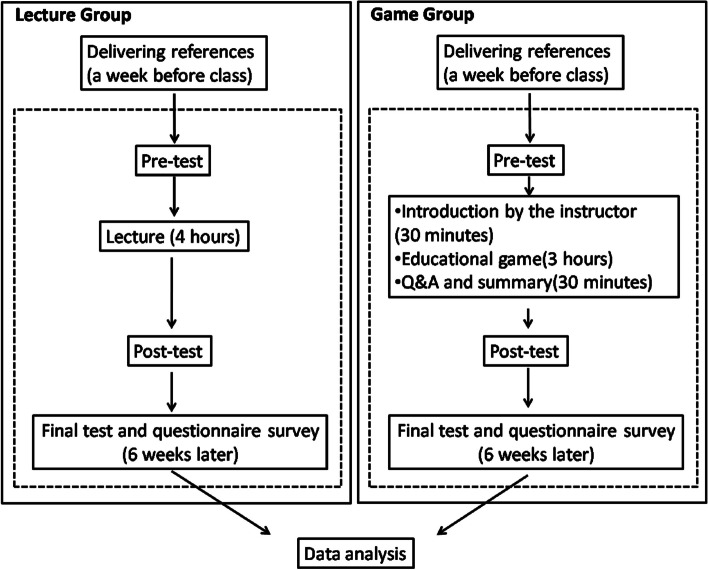
General overview of the teaching procedure. Figure Legend: The diagram shows the teaching and testing processes for the two groups of trainees. Q&A: questions and answers

In the game group, the delivery and requirements for references were the same as those in the lecture group. However, the training format differed. The game group received a half-hour lecture introducinggeneral principles, followed by 3 hours of gameplay. The last halfhour was dedicated to summarizing the course and addressing questions or concerns. Similar to the lecture group, the trainees in this group also completed pre-, post-, and final tests. Additionally, a brief survey ofthe teaching methods was conducted at the end of the final test (see Fig. 1).

### Data analysis

All data were analyzed using IBM SPSS Statistics (version 20.0;IBM Inc., Armonk, NY, USA). Only the trainees who participated in all three tests were included in the analysis. In total, there were 209 trainees, but 11 individuals (6 from the lecture group and 5 from the game group) were excluded due to incomplete data. Therefore, the data of 198 trainees were ultimately included in the analysis.

In addition, measurement data with a normal distribution were described as mean (standard deviation, SD). In contrast, measurement data with non-normal distributions were expressed as median [first quartile, third quartile]. Furthermore, enumeration data were constructed using composition ratios.

Moreover, a generalized estimating equation (GEE) was employed to compare the groups’ pre-, post-, and final test scores. The Mann–Whitney U test was used to compare the questionnaire scores between the two groups. The statistical significance was set at a level of 0.05.

## Results

Among the data included in the analysis, 97 (48.99%) participants were in the lecture group, and 101 (51.01%)were in the game group.

The number of male trainees in the lecture and game groups was 30 (30.93%) and 33 (32.67%), respectively. The mean age of participants in the lecture group was 27.44 ± 4.31 years, whereas that of the game group was 28.05 ± 4.29 years. There were no significant differences in sex or age (Table 1). Regarding the test scores, no significant differences were found between the two groups in the pre- and post-tests. However, a significant difference was observed in the final test scores conducted 6 weeks later (Table 1).

**Table 1:** Characteristics of Both the Lecture Group and the Game Group

Item	The Lecture Group (*N* = 97)	The Game Group (*N* = 101)	*p*-value
gender			
male	30 (30.93%)	33 (32.67%)	0.792
female	67 (69.07%)	68 (67.33%)	
age (years)	27.44(SD: 4.31)	28.05(SD:4.29)	0.323
test score			
pre- test	6.5 [5.5, 7]	6.5 [5.5, 7.5]	0.635
post- test	9.5 [9, 9.5]	9 [9, 9.5]	0.438
final test	8.5 [8, 9.5]	9 [8.5, 9.5]	0.001*

*SD* standard deviation

The test scores are described in the form of median [first quartile, third quartile]

* *p* < 0.05

According to the GEE analysis, the overall scores for the post-test and final test were higher compared to the pre-test scores. Additionally, there was a significant difference in the overall test scores between the two groups, with the game group demonstrating superior performance compared to the control group (odds ratio = 1.363, *p* value = 0.010). Further details of the GEE results can be found in Part 3 of the supplementary materials.

Table 2 presents the results of the questionnaire ratings for the two groups. The median [first quartile, third quartile] of the learning motivation questionnaire ratings were 4 [3, 4] for the lecture group and 5 [4, 5] for the game group. There were significant differences between the questionnaire ratings of the two groups (*p* < 0.001), indicating that the game group had higher learning motivation for the learning activity.

**Table 2:** Results of the questionnaire ratings of the two groups

Items	Median of the questionnaire ratings		*p* value
	Lecture group	Game group	
learning motivation	4 [3, 4]	5 [4, 5]	<0.001*
cognitive load	4 [4, 5]	3 [3, 4]	<0.001*
mental effort	4 [4, 5]	3 [2, 3]	<0.001*
mental load	4 [3, 4]	4 [3, 4]	0.539
perceived usefulness	/	5 [5, 6]	/
perceived ease of use	/	5 [5, 6]	/

In Table 2, the questionnaire ratings are described in the form of median [first quartile, third quartile]

* p < 0.05

The median [first quartile, third quartile] of the overall cognitive load ratings were 3 [3, 4] and 4 [4, 5] for the game and lecture groups, respectively. There was a significant difference between the cognitive load ratings of the two groups (*p* < 0.001).

This study further compared two aspects of cognitive load: mental load and mental effort. The median [first quartile, third quartile] for the mental effort dimension were 3 [2, 3] and 4 [4, 5] for the game and lecture groups, respectively (p < 0.001). For mental load, the median [first quartile, third quartile] were 4 [3, 4] and 4 [3, 4] for the game and lecture groups, respectively. There was no significant difference in the mental load ratings between the two groups (*p* = 0.539).

To better understand the trainees’ perceptions of the use of the serious game, this study collected the feedback of the trainees in the game group regarding “perceived usefulness” and “perceived ease of use,” as shown in Table 2. Most trainees provided positive feedback on the two dimensions of the serious game.

## Discussion

To the best of our knowledge, this IEMT training game is the first serious game intended for newly joined nurses of IEMTs. Therefore, this study presents an initial investigation into the applicability of serious games.

Both lectures and serious games improved post-class test scores to the same level, consistent with previous studies. Krishnan et al. found that an educational game on hepatitis significantly improved knowledge scores [20]. Additionally, our study showed higher knowledge retention in the game group after 6 weeks, in line with previous studies on serious games. In a study on sexually transmitted diseases, game-based instruction was found to improve knowledge retention for resident physicians compared to traditional teaching methods [21]. The IEMTtraining game, designed as a role-playing game, is more likely to enhance knowledge retention in newly joined nurses in the long term. Therefore, serious games should be included in the teaching of IEMT training.

This study demonstrated improved learning motivation in the game group, consistent with previous research indicating that game-based learning enhances motivation due to the enjoyable and challenging nature of the games [22, 23]. A systematic review by Allan et al. further supports the positive impact of game-based learning tools on the motivation, attitudes, and engagement of healthcare trainees [24].

As serious games are a novel learning experience for trainees, it is worth investigating the cognitive load they experience. Our study found that serious games effectively reduce trainees’ overall cognitive load, particularly in terms of lower mental effort. Mental effort refers to the cognitive capacity used to handle task demands, reflecting the cognitive load associated with organizing and presenting learning content, as well as guiding student learning strategies [25, 26]. This reduction in cognitive load is a significant advantage of serious gaming, as it helps learners better understand and organize their knowledge. However, our study did not find a significant difference in mental load between the two groups. Mental load considers the interaction between task and subject characteristics, based on students’ understanding of tasks and subject characteristics [18]. This finding is intriguing as it aligns with similar observations in game-based education for elementary and secondary school students [27], but is the first mention of game-based education in academic papers related to nursing training.

In our survey of the game group participants, we found that their feedback regarding the perceived ease of use and usefulness of the game was overwhelmingly positive. This indicates that the designed game was helpful to learners during the learning process. Moreover, the game’s mechanics were easily understood by the trainees without requiring them to investsignificant time and effort to understand the game rules and controls.

This study had some limitations. First, this retrospective observational study may have been susceptible to sampling bias due to the non-random grouping of trainees. It only reviewed existing data from the training database, and future research should be conducted to validate our findings through prospective studies. Therefore, randomized controlled trials are required. Second, the serious game is currently available only in China. We are currently developing an English version to better align with the training requirements of international IEMT nurses. Third, the development of such serious gamescan be time-consuming. To address this problem, we propose a meta-model to help researchers and instructors select appropriate game development models to implement effective serious games.

## Conclusion

An IEMT training game for newly joined nurses is a highly promising training method. Its potential lies in its ability to offer engaging and interactive learning experiences, thereby effectively enhancing the training process. Furthermore, the game improved knowledge retention, increased motivation to learn, and reduced cognitive load. In addition, the game’s mechanics are easily understood by trainees, which further enhances its effectiveness as a training instrument.

### Supplementary Information

**Supplementary Material 1.**

## Data Availability

Availability of data and materials can be ensured through direct contact with the author. If you require access to specific data or materials mentioned in a study or research article, reaching out to the author is the best way to obtain them. By contacting the author directly, you can inquire about the availability of the desired data and materials, as well as any necessary procedures or restrictions for accessing them. Authors are willing to provide data and materials to interested parties. They understand the importance of transparency and the positive impact of data sharing on scientific progress. Whether it is raw data, experimental protocols, or unique materials used in the study, authors can provide valuable insights and resources to support further investigations or replications. To contact the author, one can refer to the email address provided in the article.

## Abbreviations

WHO: World Health Organization
IEMT: International Emergency Medical Team
MDS: Minimum Data Set
GEE: Generalized estimating eq.
SD: Standard deviation

## References

[1] World Health Organization.Classification and minimum standards for emergency medical teams. https://apps.who.int/iris/rest/bitstreams/1351888/retrieve. Published 2021. Accessed May 6, 2023.

[2] World Health Organization. Classification and Minimum Standards for Foreign Medical Teams in Sudden Onset Disasters. https://cdn.who.int/media/docs/default-source/documents/publications/classification-and-minimum-standards-for-foreign-medical-teams-in-suddent-onset-disasters65829584-c349-4f98-b828-f2ffff4fe089.pdf?sfvrsn=43a8b2f1_1&download=true. Published 2013. Accessed May 6, 2023.

[3] Brunero S, Dunn S, Lamont S (2021). Development and effectiveness of tabletop exercises in preparing health practitioners in violence prevention management: a sequential explanatory mixed methods study. Nurse Educ Today.

[4] Sena A, Forde F, Yu C, Sule H, Masters MM (2021). Disaster preparedness training for emergency medicine residents using a tabletop exercise. Med Ed PORTAL.

[5] Moss R, Gaarder C (2022). Exercising for mass casualty preparedness. Br J Anaesth.

[6] Hu H, Liu Z, Li H (2022). Teaching disaster medicine with a novel game-based computer application: a case study at Sichuan University. Disaster Med Public Health Prep.

[7] Chi CH, Chao WH, Chuang CC, Tsai MC, Tsai LM (2001). Emergency medical technicians' disaster training by tabletop exercise. Am J Emerg Med.

[8] Hu H, Lai X, Li H (2022). Teaching disaster evacuation management education to nursing students using virtual reality Mobile game-based learning. Comput Inform Nurs.

[9] van Gaalen AEJ, Brouwer J, Schönrock-Adema J (2021). Gamification of health professions education: a systematic review. Adv Health Sci Educ Theory Pract.

[10] Adjedj J, Ducrocq G, Bouleti C (2017). Medical student evaluation with a serious game compared to multiple choice questions assessment. JMIR Serious Games.

[11] Hu H, Xiao Y, Li H (2021). The effectiveness of a serious game versus online lectures for improving medical Students' coronavirus disease 2019 knowledge. Games Health J.

[12] Pimentel J, Arias A, Ramírez D (2020). Game-based learning interventions to Foster cross-cultural care training: a scoping review. Games Health J.

[13] Hu H, Lai X, Yan L (2021). Improving nursing Students' COVID-19 knowledge using a serious game. Comput Inform Nurs.

[14] Menin A, Torchelsen R, Nedel L (2018). An analysis of VR technology used in immersive simulations with a serious game perspective. IEEE Comput Graph Appl.

[15] Kubo T, Chimed-Ochir O, Cossa M (2022). First activation of the WHO emergency medical team minimum data set in the 2019 response to tropical cyclone Idai in Mozambique. Prehosp Disaster Med.

[16] Jafar AJN, Sergeant JC, Lecky F (2020). What is the inter-rater agreement of injury classification using the WHO minimum data set for emergency medical teams?. Emerg Med J.

[17] Hwang GJ, Chang HF (2011). A formative assessment-based mobile learning approach to improving the learning attitudes and achievements of students. Comput Educ.

[18] Hwang G-J, Yang L-H (2013). Sheng-yuan Wang.Concept map-embedded educational computer game for improving students’ learning performance in natural science courses. Comput Educ.

[19] Chu HC, Hwang GJ, Tsai CC (2010). A two-tier test approach to developing location-aware mobile learning system for natural science course. Comput Educ.

[20] Krishnan S, Blebil AQ, Dujaili JA, Chuang S, Lim A (2023). Implementation of a hepatitis-themed virtual escape room in pharmacy education: A pilot study. Educ Inf Technol (Dordr).

[21] Butler SK, Runge MA, Milad MP (2020). A game show-based curriculum for teaching principles of reproductive infectious disease (GBS PRIDE trial). South Med J.

[22] Haruna H, Hu X, Chu SKW (2018). Improving sexual health education programs for adolescent students through game-based learning and gamification. Int J Environ Res Public Health.

[23] Rewolinski JA, Kelemen A, Liang Y (2020). Type I diabetes self-management with game-based interventions for pediatric and adolescent patients. Comput Inform Nurs.

[24] Allan R, McCann L, Johnson L, Dyson M, Ford J (2023). A systematic review of 'equity-focused' game-based learning in the teaching of health staff. Public Health Pract (Oxf).

[25] Zumbach J, Rammerstorfer L, Deibl I (2020). Cognitive and metacognitive support in learning with a serious game about demographic change. Comput Hum Behav.

[26] Chang C-C, Liang C, Chou P-N (2017). Is game-based learning better in flow experience and various types of cognitive load than non-game-based learning? Perspective from multimedia and media richness. Comput Hum Behav.

[27] Kalmpourtzis G, Romero M (2020). Constructive alignment of learning mechanics and game mechanics in serious game design in higher education. Int J Serious Games.

