# Supplementary material for: Training nurses in an international emergency medical team using a serious role-playing game: a retrospective comparative analysis
Source: BMC Med Educ. 2024 Apr 22;24:432. doi: 10.1186/s12909-024-05442-x (PMC11034038; doi:10.1186/s12909-024-05442-x)
Supplement: Supplementary file 1 — Supplementary Material 1. [file 12909_2024_5442_MOESM1_ESM.pdf]

Supplemental Digital Content

Part1

Introduction of the EMT-training game

Fig.1. The 2D Game Scene

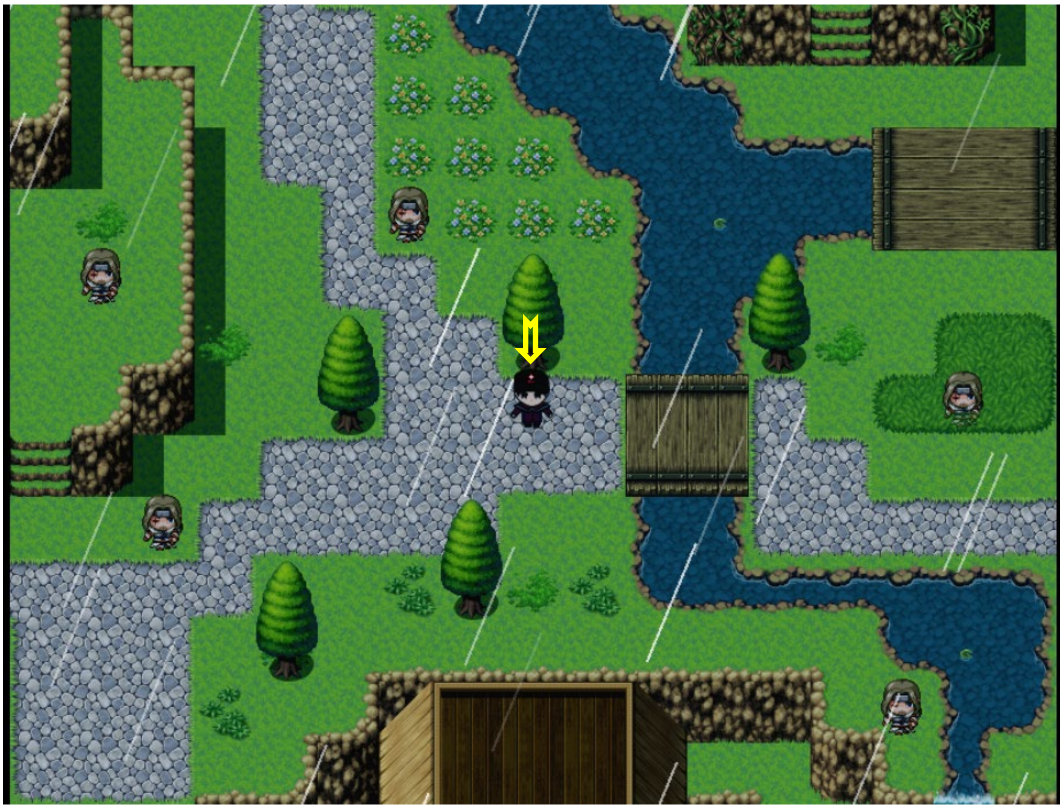

The yellow arrow points to the player.

In the game, the mouse controls the movement of game characters. In addition, players can click on the injured (non player character) to view the condition of the injury and provide treatment.

Fig.2. The dialog interface

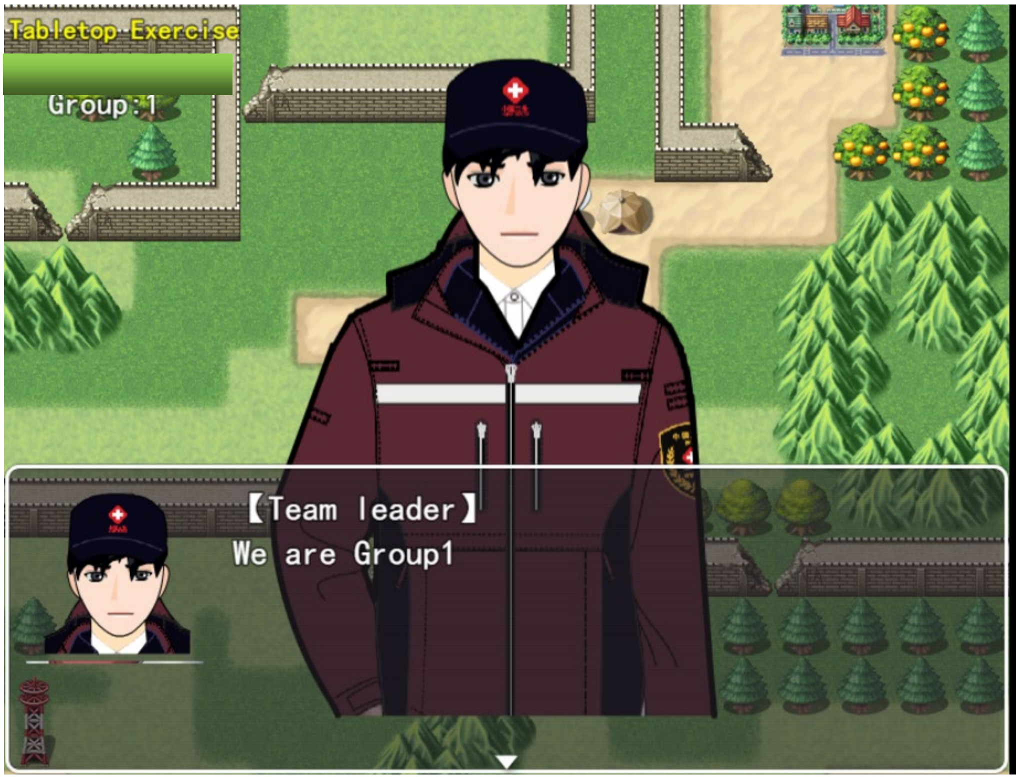

Fig.3.Viewing the injured persons injury information

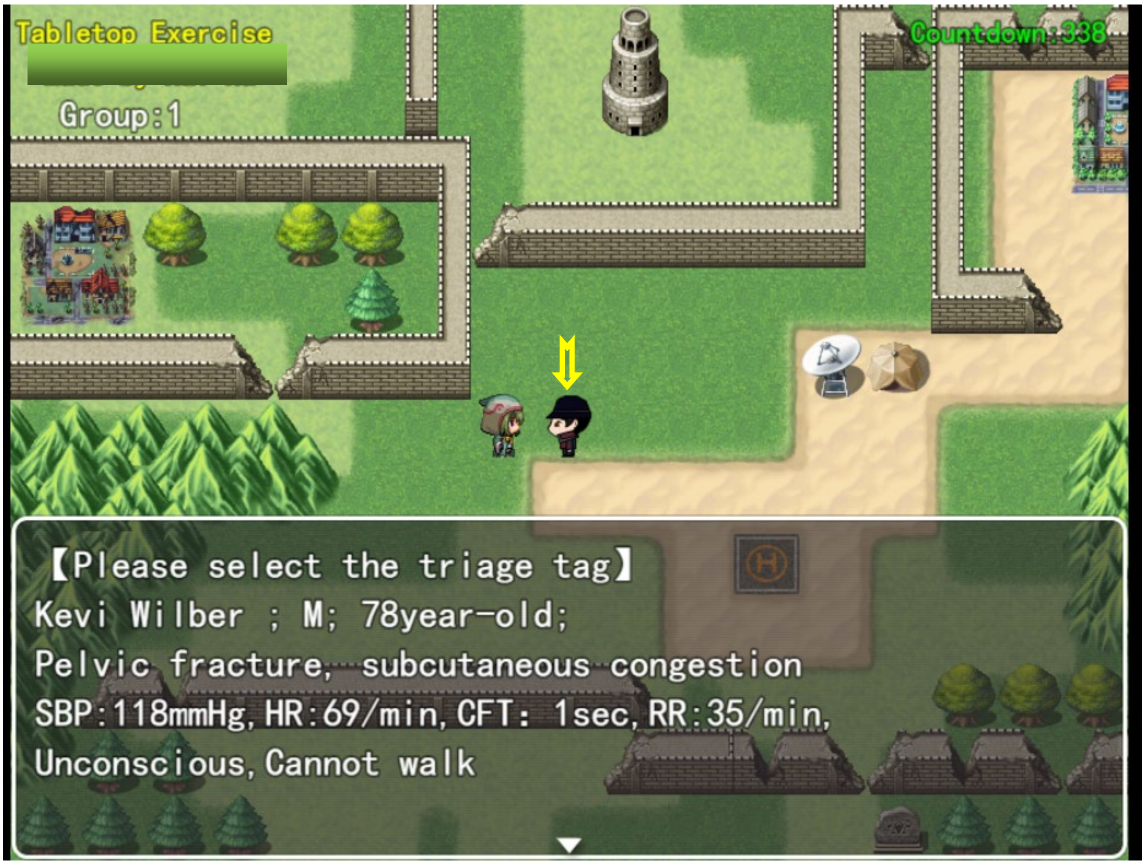

Fig.4.Selecting triage tags for the injured persons

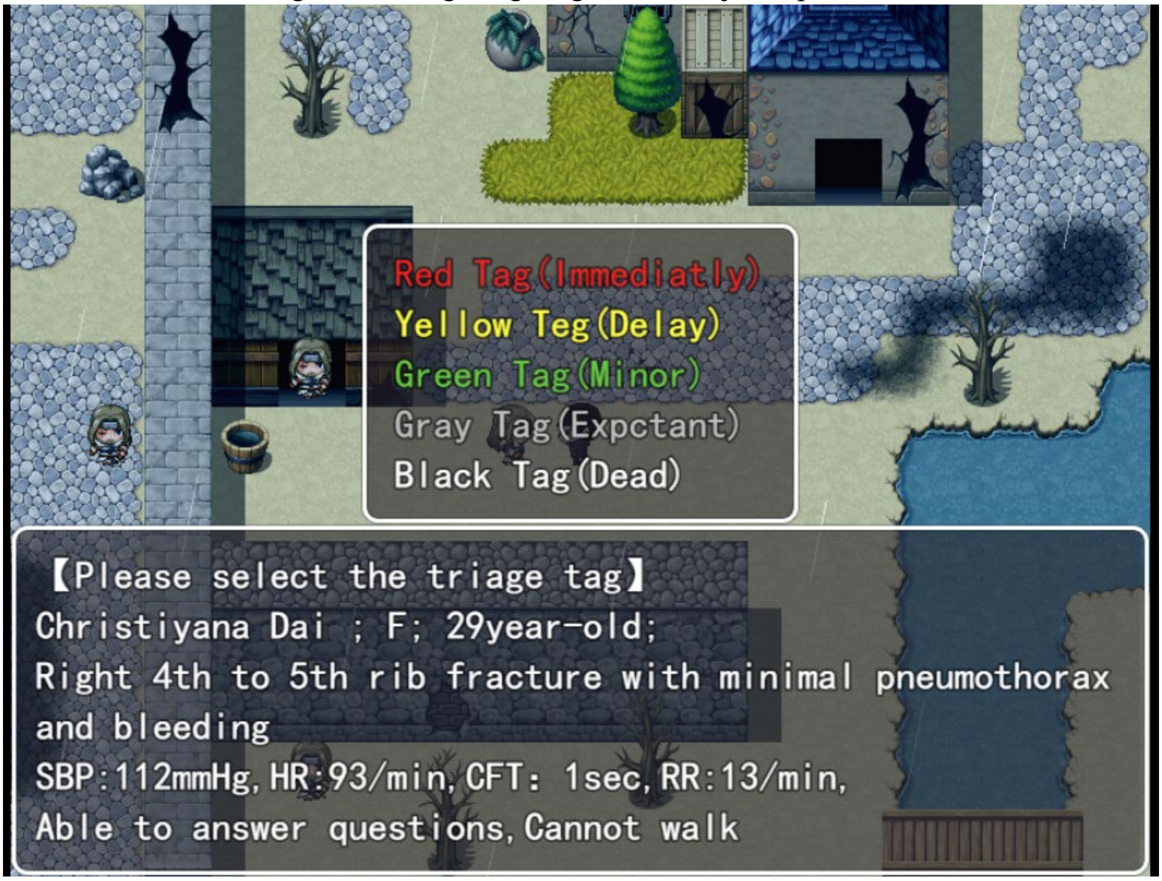

Fig.5.Selecting the necessary medical supplies from the kit to provide treatment

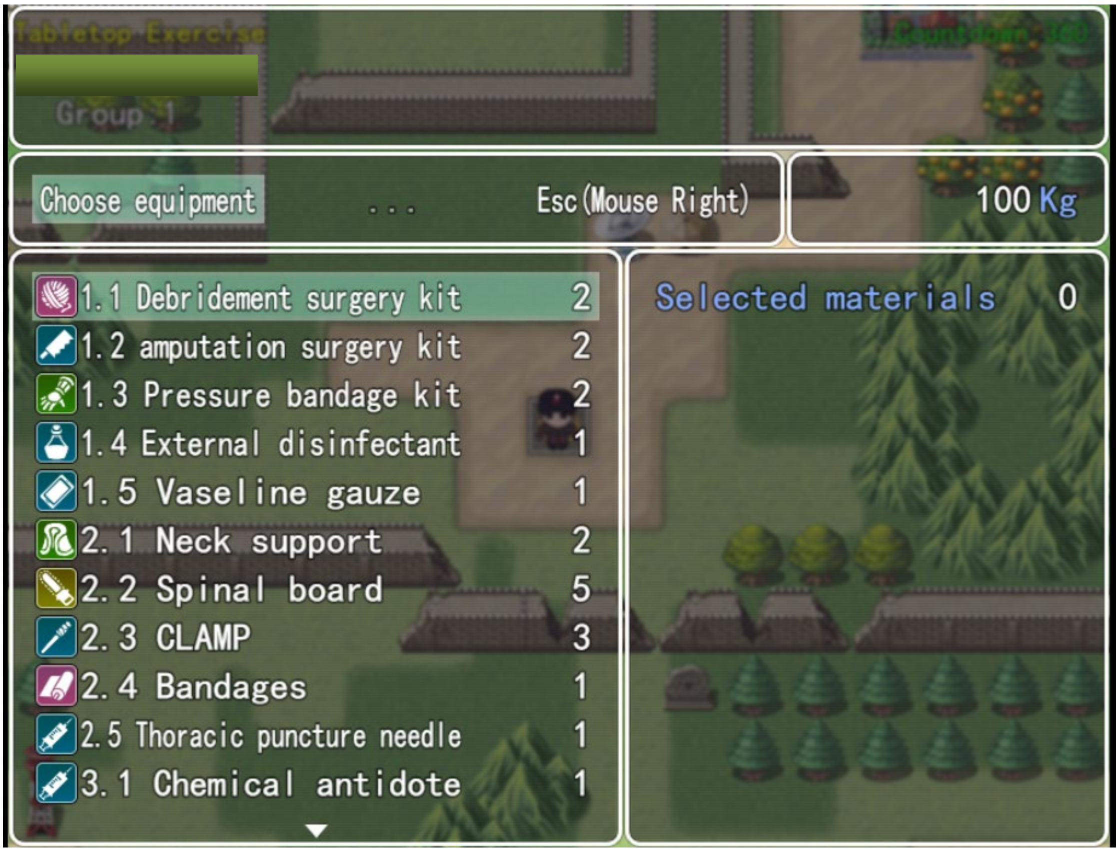

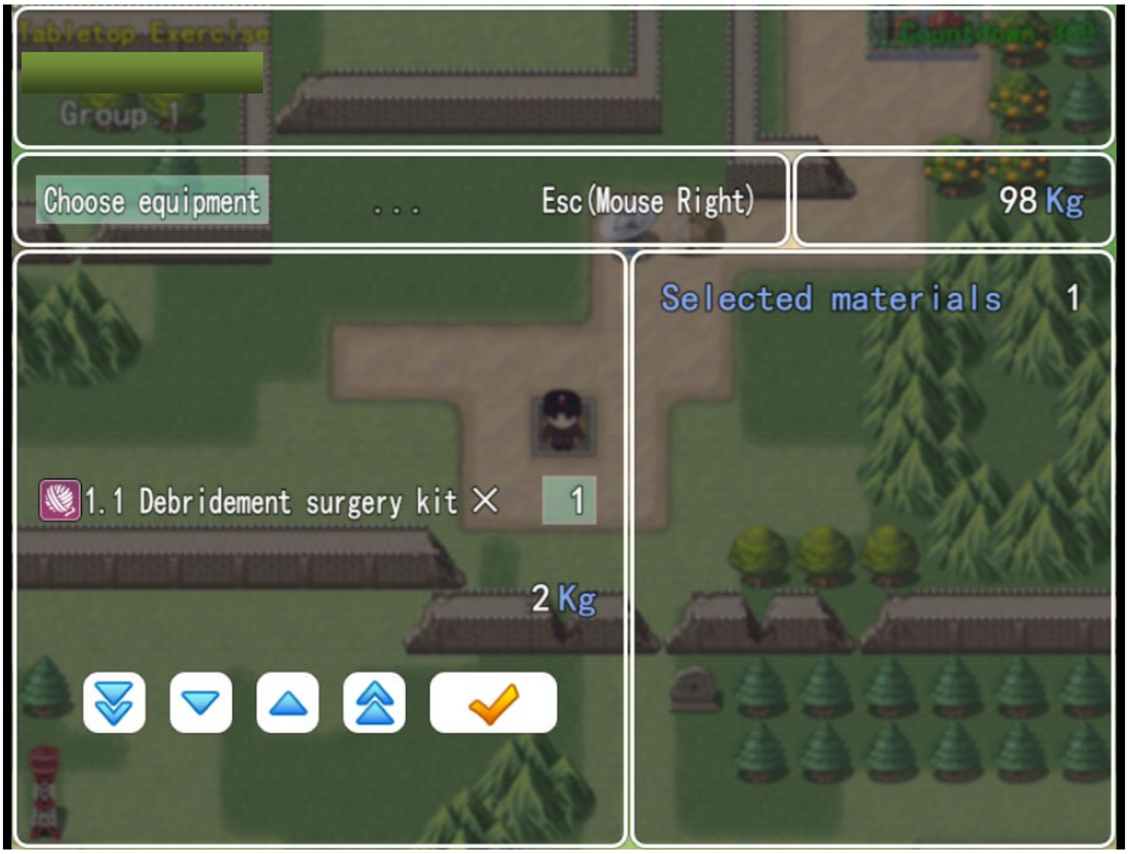

Fig.6.Reporting injury information following the minimum data set designed by the WHO

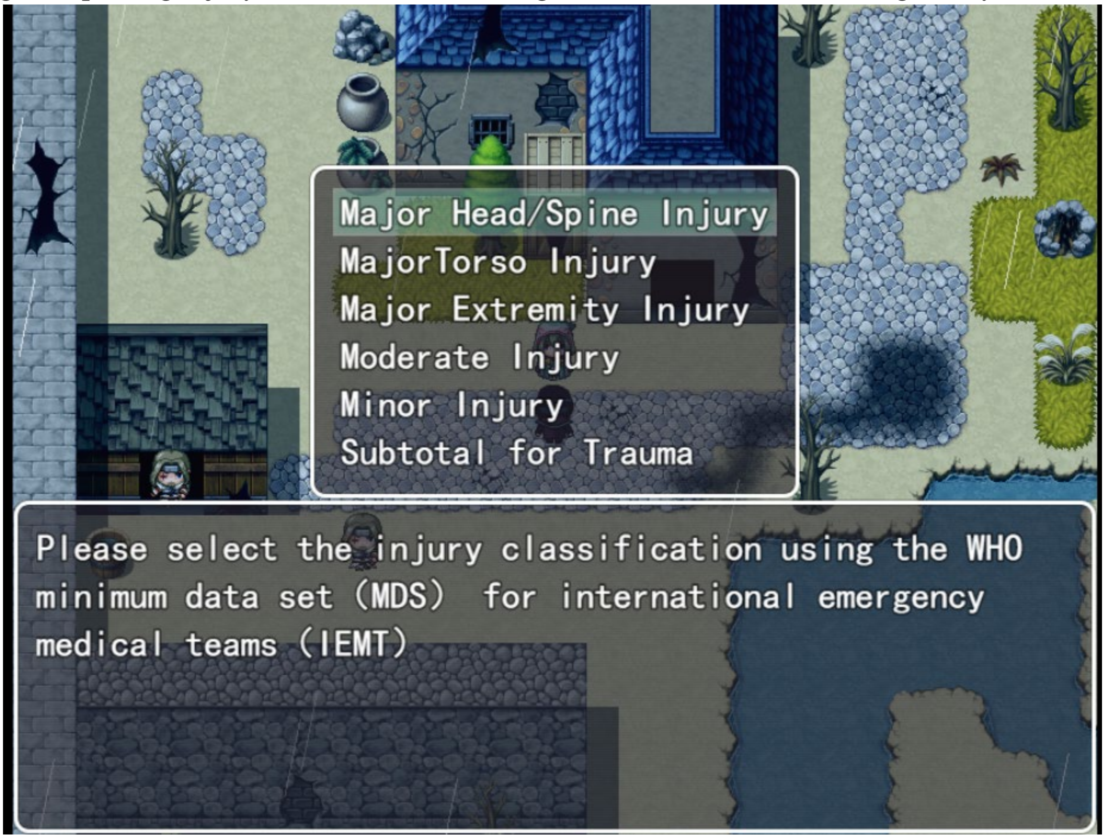

Part2

The items of the questionnaires utilized in this study

This survey employs the Likert scale, with a rating scale from 1 to 6. A score of 6 indicates strong agreement, while a score of 1 represents strong disagreement.

Learning motivation

1. I think learning the course content is interesting and valuable.
2. I would like to learn more and observe more in the course.
3. It is worth learning the course content.
4. It is important for me to learn the course well.
5. It is important to know the knowledge related to EMT.
6. I will actively search for more information and learn about the course content.
7. It is important for us to take the course.

Cognitive load

Mental load

1. The learning content in this learning activity was difficult for me.
2. I had to put a lot of effort into answering the questions in this learning activity.
3. It was troublesome for me to answer the questions in this learning activity.
4. I felt frustrated answering the questions in this learning activity.
5. I did not have enough time to answer the questions in this learning activity.

Mental effort

1. During the learning activity, the way of instruction or learning content presentation caused me a lot of mental effort.
2. I need to put lots of effort into completing the learning tasks or achieving the learning objectives in this learning activity.
3. The instructional way in the learning activity was difficult to follow and understand.

Technology acceptance

Usefulness

1. The learning approach enriched the learning activity.
2. The learning system was helpful to me in acquiring new knowledge.
3. The learning mechanisms provided by the learning system smoothed the learning process.
4. The learning system helped me obtain useful information when needed.
5. The learning approach helped me learn better.
6. The learning approach is more useful than the conventional learning approaches.

Ease of use

1. It is not difficult for me to learn to operate the learning system.
2. It only took me a short time to fully know how to use the learning system.
3. The learning activity conducted in the learning system was easy to understand and follow.
4. I quickly learned to use the learning system.
5. It was not difficult for me to use the learning system during the learning activity.
6. I felt that the interface of the learning system was easy to use.
7. To sum up, the learning system adopted in this learning activity was easy to learn and use.

Part3

Table1 The Results of Generalized Estimation Equation

	Coef.	S.E.	p value	OR	95%CI of OR
final test	5.035	0.148	<0.001*	153.754	115.117-205.358
post-test	5.970	0.122	<0.001*	391.387	308.382-496.734
pre-test	Ref.				
Game group	0.309	0.119	0.010*	1.363	1.078-1.722
Lecture group	Ref.				

Coef.: Coefficient;

S.E.: standard error;

OR: odds ratio;

95%CI: 95% confidence interval.

Ref.: reference;

* p< 0.05.
